# Genetic evidence of a recent Tibetan ancestry to Sherpas in the Himalayan region

**DOI:** 10.1038/srep16249

**Published:** 2015-11-05

**Authors:** Sushil Bhandari, Xiaoming Zhang, Chaoying Cui, Bianba, Shiyu Liao, Yi Peng, Hui Zhang, Kun Xiang, Hong Shi, Ouzhuluobu, Baimakongzhuo, Gonggalanzi, Shimin Liu, Gengdeng, Tianyi Wu, Xuebin Qi, Bing Su

**Affiliations:** 1 State Key Laboratory of Genetic Resources and Evolution, Kunming Institute of Zoology, Chinese Academy of Sciences, Kunming 650223, China; 2 High Altitude Medical Research Center, School of Medicine, Tibetan University, Lhasa 850000, China; 3 National Key Laboratory of High Altitude Medicine, High Altitude Medical Research Institute, Xining 810012, China; 4 Kunming College of Life Science, University of Chinese Academy of Sciences, Beijing 100049, China; 5 Institute of Primate Translational Medicine, Kunming University of Science and Technology, Kunming, Yunnan, 650500, China

## Abstract

Sherpas living around the Himalayas are renowned as high-altitude mountain climbers but when and where the Sherpa people originated from remains contentious. In this study, we collected DNA samples from 582 Sherpas living in Nepal and Tibet Autonomous Region of China to study the genetic diversity of both their maternal (mitochondrial DNA) and paternal (Y chromosome) lineages. Analysis showed that Sherpas share most of their paternal and maternal lineages with indigenous Tibetans, representing a recently derived sub-lineage. The estimated ages of two Sherpa-specific mtDNA sub-haplogroups (C4a3b1 and A15c1) indicate a shallow genetic divergence between Sherpas and Tibetans less than 1,500 years ago. These findings reject the previous theory that Sherpa and Han Chinese served as dual ancestral populations of Tibetans, and conversely suggest that Tibetans are the ancestral populations of the Sherpas, whose adaptive traits for high altitude were recently inherited from their ancestors in Tibet.

Following the groundbreaking ascent of Mount Everest by Tenzing Norgay and Sir Edmund Hillary, the Sherpa people gained such cachet that their name became vernacular shorthand and pop culture reference for much of the twentieth century. Curiously, despite the hypoxic conditions of their highland homes in the Khumbu region of Nepal and smaller settlements along the Sino-Nepalese border in the Tibet Autonomous Region of China in Dingjie County and Zhangmu Town, Sherpas seem to cope well with the environments and avoid altitude sickness. This heightened adaptation to high altitude hypoxic environments seems to suggest that the Sherpas have been living in the region for a relatively long time and acquired an inherited adaptation. Unfortunately, archaeological evidence from the Himalayas that might verify this hypothesis is quite limited. The existing archaeological data suggests that the first peoples arrived at the Tibetan plateau as early as 30,000 years ago and were later followed by successive migrations from different times and places, ultimately creating a complex mosaic of population history at the Himalayas^1^. Specifically, some stone tools in western Tibet were dated to 25,000 to 20,000 years ago and were claimed to be similar to other stone tools excavated in Nepal, hinting at a northward migration into the Tibetan plateau from the southern Himalayas^1^,^2^.

Some genetic evidence also supports the Nepal-Tibet migrations, though in different patterns. A recent genetic study went so far as to posit that Sherpas and Han Chinese were the two ancestral populations of modern Tibetans, and consequently the genetic adaptation to high altitude hypoxia seen among Tibetans was likely inherited from the ancestral Sherpas^3^. Conversely, a recent study of mtDNA diversity on a small Sherpa sample from Zhangmu Town in Tibet reported two Sherpa-specific haplotypes with relatively young ages (<1,500 years), suggesting a Tibet into Nepal migration granting the Sherpas’ well-known adaptation at high altitudes^4^. The disparity in these two views reflects a fundamental divide in the evolution of adaptive features among Sherpas, holding to either a short (<1500 years) or long (30,000 years) duration of colonization at high altitude. Complicating this controversy is the limited genetic studies of populations from the southern Himalayas, leaving the answer of when and where the Sherpa people originated and settled at the high altitude regions of Nepal unclear.

In the present study, we analyzed Y-chromosome and mtDNA diversity of 582 unrelated Sherpa samples collected from different places in both Nepal and Tibet (Fig. 1). We genotyped the Y chromosomal single nucleotide polymorphisms (Y-SNPs) and Y short tandem repeats (Y-STRs) from 277 male Sherpa samples. We also sequenced the complete mtDNA genomes of 89 Sherpa individuals and genotyped the remaining samples based on the control region (HVS-I and HVS-II) and partial coding regions of mtDNA. Data from both the paternal (Y chromosome) and the maternal (mtDNA) markers indicates that the Sherpa people are a recently derived sub-lineage of Tibetans, supporting a short colonization and settlement in the southern Himalayas.

## Results

### Y-chromosomal diversity in Sherpa population

**Figure 1:**
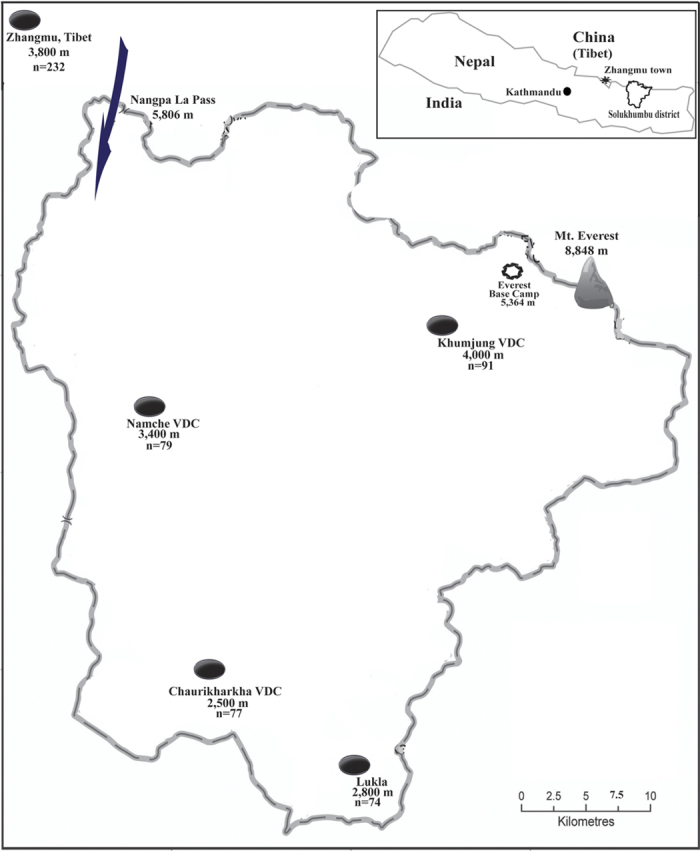
Sampling locations of Sherpa populations in Nepal and Tibet. The altitudes of the locations (dark spots) range from 2,500 m–4,000 m. The proposed Tibet-Nepal migratory route of the Sherpa ancestors through Nangpa La Pass is indicated with an arrow. The map was created using Surfer10 (Golden Software Inc., Golden, USA), and the figure was generated using Microsoft Powerpoint 2011 (Microsoft Corporation, USA).

**Figure 2:**
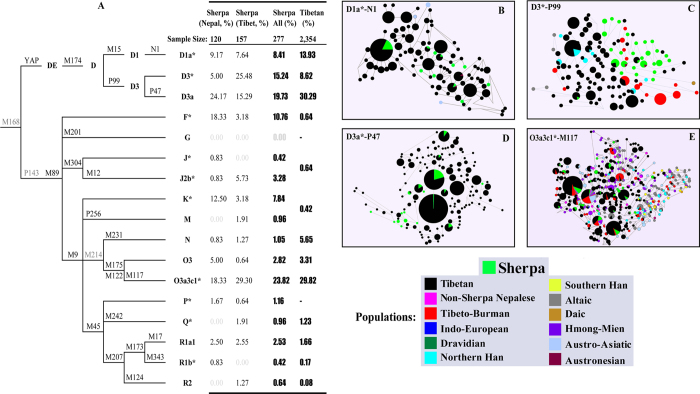
Comparison of Y chromosome diversity among Sherpas, Tibetans and other Asian populations. (**A**) Phylogenetic tree of the Y chromosome haplogroups and their frequency distributions in Sherpas and Tibetans. (**B**–**E**) The Y-STR networks of the four major haplogroups showing the distributions of STR haplotypes in Sherpas and other Asian populations. Populations were labeled with different colors based on their belonged language families.

In total, DNA samples of 582 unrelated Sherpa individuals representing two geographic populations from Nepal (350 individuals) and Tibet (232) were collected along with samples of 90 non-Sherpa Nepalese from Solukhumbu district of Nepal (Fig. 1). Using the current phylogeny of the human Y-chromosomes, we assigned the samples from 277 Sherpa males into four major Y-chromosomal haplogroups including D-M174 (44.04%), O-M175 (27.08%), F-M89 (9.75%), and K*M9 (7.22%) (Fig. 2A). Of these groups, D-M174 and O-M175 are also the two dominant Y-haplogroups in Tibetans (52.84% and 33.13% respectively)^5^, suggesting a close paternal relation between Sherpas and Tibetans. Likewise, we observed several rare (0.36–3.61%) haplogroups in Sherpas (G-M201, J-M304, M-P256, N-M231, P-M45, Q-M242 and R-M207) that are also seen among Tibetans in similarly low frequencies (Fig. 2A). Due to the similarities in the distributions of Y-haplogroups between Sherpas from Nepal and those from Tibet, the data were merged together for further analyses.

Comparing Sherpas, Tibetans, and Han Chinese showed that the D-M174 is the predominant haplogroup in Sherpas (43.38%) and prevalent in Tibetans (52.84%)^5^, but rare among both Han Chinese (1.4–6.51%)^6^,^7^ and other Asian populations (0.02–0.07%)^8^, aside from Japanese (34.7%) who possesses a distinct D-M174 lineage highly diverged from those in Tibetans and other Asian populations^9^,^10^. Among Tibetans there are five D-M174 sub-haplogroups (D*-M174, 0.08%; D1*-M15, 1.40%; D1a-N1, 13.93%; D3*-P99, 8.62%; D3a-P47, 30.29%), three of which are also present among Sherpas (D1a-N1, 8.41%; D3*-P99, 15.24%; D3a-P47, 19.73%; see Fig. 2A). To explore the structure of the D-M174 sub-haplogroups, we constructed the Y-STR network of these sub-lineages and observed that the Y-STR haplotypes among Sherpas represent a subset of those found in Tibetans, implying that the paternal lineages of Sherpas were likely derived from those in Tibetans (Fig. 2B–D). Moreover, there were Sherpa-specific Y-STR haplotypes under the D3*-P99 sub-haplogroup closely linked with those in Tibetans (Fig. 2C), implying a shallow divergence between Sherpas and Tibetans at this paternal lineage.

A similar pattern of Y-STR network was also seen among the second most common haplogroup O3a3c1*-M117 in Sherpas (23.82%), which is also present in Tibetans (29.82%), Han Chinese (9.6–16.3%) and many other East Asian and Southeast Asian populations (5.5–16%)^5^, but Sherpas shared most of their Y-STR haplotypes of O3a3c1-M117 with Tibetans (Fig. 2E).

Taken together, D-M174 and O-M175 account for 71.12% of the paternal lineages in Sherpas, bolstering the case for Tibetans as the ancestral population of Sherpas. Likewise, the relative rarity of other Y haplogroups (<10%) among Sherpas (supplementary Figure S1) were also rare among Tibetans, and mostly absent in other East Asian populations. Most of these haplogroups are prevalent in India (F*-M89, J2b*-M12 and R1a1-M17) and Island Southeast Asia (K*-M9), suggesting either shared ancient Y-chromosome lineages or limited recent admixture of Sherpas with surrounding populations.

### Mitochondrial DNA diversity in Sherpas

**Figure 3:**
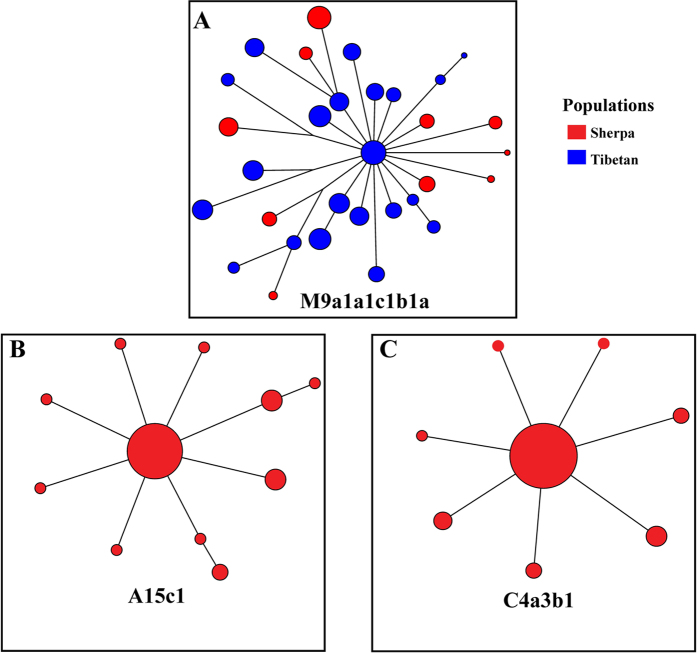
Networks of three mtDNA sub-haplogroups (A: M9a1a1c1b1a1; B: A15c1; C: C4a3b1) among Sherpas and Tibetans. The star-like networks suggest recent population expansion. The complete mtDNA genome sequences were used to construct the networks.

Classification of mtDNA haplogroups of 582 Sherpa samples yielded several major haplogroups including A (27.15%), M9a (24.23%), C4a (20.96%), M70 (7.22%) and D (5.84%) (Table 1), which together cover 85.40% of the tested Sherpas. Among Tibetans these same groups A (14.63%), M9a (22.48%) and D (16.53%) also constituted the major haplogroups^5^. M9a and its four sub-haplogroups (M9a1a, M9a1a2, M9a1b1and M9a1a1c1b1a) are widely distributed in East Asia and Southeast Asia^11^,^12^, though M9a1a1c1b1a is quite rare among most Asian populations but among Sherpas is the most common (58.16% of the total M9a individuals) and among Tibetans highly prevalent (58.06% of the total M9a individuals)^12^. The shared prevalence of M9a1a1c1b1a in Sherpas and Tibetans suggests a close maternal relationship between them. After constructing a network using 36 complete mtDNA genome sequences (34 previously published and two of present study) from 13 Sherpas and 23 Tibetans belonging to M9a1a1c1b1a, we observed that all the Sherpa individuals lie at the tip branches, suggesting they likely derived from the Tibetan core haplotype (Fig. 3A). Interestingly, the star-like topology (*i.e.* a core haplotype surrounded by other haplotypes with only one or two mutation steps away from the core) suggests a relatively recent expansion of this lineage, consistent with a previous observation made on Tibetans^5^. The close genetic affinity between the two populations is further supported by Haplogroup D among Sherpas (5.84%), which was also widely found in Tibetans^5^. But more importantly M70—previously considered a Tibetan specific lineage^13^,^14^—was also present among Sherpas (7.22%) (Table 1). Much like the observed pattern of the Y-haplogroups, the Sherpa mtDNA haplogroup composition bears strong similarity with that observed among Tibetans.

Interestingly, two Sherpa-specific sub-haplogroups were not detected in Tibetans or other Asian populations: A15c1 (17.27%), a sub-lineage under Haplogroup A15c, and C4a3b1 (21.82%), a sub-lineage under C4a3b^4^. These two Sherpa-specific sub-haplogroups together account for nearly 40% of the maternal lineages of Sherpas. Sequencing the entire mitochondrial genomes of 79 Sherpa individuals belonging to these two sub-haplogroups showed an interesting sequence variation pattern. Consistent with the previous report^4^, A15c1 defined by four mutations (T4216C, A9052G, T13111C and A15924G), and C4a3b1 by other four mutations (G3745A, 5899insC, C11155T, A13563G) only exist in Sherpas, but their mother haplogroups (A15c and C4a3b) are present in both Tibetans and Han Chinese^15^,^16^ (supplementary Figure S2). Further network analysis showed that A15c1 and C4a3b1 both form a star-like structure, denoting recent lineage expansion among Sherpas (Fig. 3B,C). We estimated the coalescence ages of A15c1 (1500 years ago) and C4a3b1 (940 years ago), both of which are similar with the previous estimates^4^ and consistent with the proposed recent expansion. The young ages of these two Sherpa-specific sub-haplogroups hint at a recent bottleneck during migration and latter population expansion of Sherpas, which fits our observation of a shallow divergence of the Y chromosome haplogroup D3*-P99 between Sherpas and Tibetans.

Of the other rare mtDNA haplogroups in Sherpas—Z (2.75%), F (2.58%), M13 (1.72%) and U (1.37%), as well as those with <1% frequencies (M3, M5, W, G, M10, H, M62, M11a, M38, M61 and M74)—a majority including G, Z, F, M62, M10, M11a, M13, and F have been seen among Tibetans. By contrast, the presence of the other haplogroups M5, M3, W, M38, M61, M74 and U among Sherpas may indicate minor gene flows from surrounding South Asian populations (see Table 1).

### Genetic relationship of Sherpas with other Asian populations

**Figure 4:**
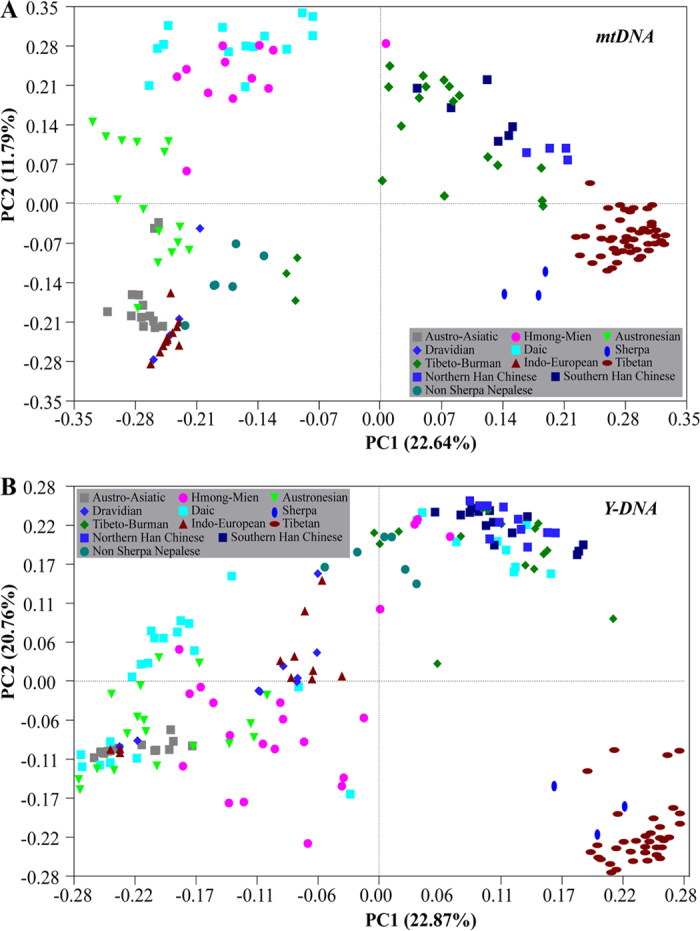
Maps of principal component analysis (PCA) based on mtDNA and Y-Chromosome haplogroup frequencies among Sherpas, Tibetans and other Asian populations.

We explored the genetic relationship between Sherpas and other Asian populations, and principal component analysis (PCA) of both mtDNA and Y chromosome haplotype frequencies displayed a close relationship between Sherpas and Tibetans (Fig. 4A,B). In particular, the PCA map of the Y chromosome shows Sherpas mingled together with Tibetan populations and forming a separate cluster from other Asian populations (Fig. 4B). The PCA map of mtDNA further places the Sherpas relatively closer to Tibetans compared to other Asian populations but form a separate cluster, implying a shallow divergence from Tibetans likely due to the recent expansions of the two Sherpa-specific mtDNA sub-haplogroups (A15c1 and C4a3b1) (Fig. 4A). By contrast, non-Sherpa Nepalese are only distantly related to Sherpas, clustering with populations from the Indian subcontinent and East Asia, suggesting limited gene flows between Sherpas and non-Sherpas in the markedly small area of Nepal nestled between India and China.

## Discussions

Our investigation of the Sherpas’ origins via analysis of Y-chromosome and mtDNA diversity analysis supports a close genetic relationship between Sherpas and Tibetans. Furthermore, the detailed genetic diversity pattern revealed in the Y-STR network and the mtDNA phylogenetic trees indicate that Sherpas represent a recently derived lineage from Tibetans dated to less than 1,500 years ago. Further BSP analysis sketches a general picture of a recent bottleneck of the Sherpa population size less than 2,000 years ago (supplementary Figure S3), consistent with the proposed recent migration of the Sherpa ancestors from Tibet. These genetic findings further bolster the established linguistic affiliation among the speakers of the Tibeto-Burman language subfamily within the Sino-Tibetan family (the other subfamily is Han). Collectively, these results concur with the previous hypothesis positing the migration of the Baric branch of the Tibeto-Burman languages from the Tibetan plateau to Nepal following their dispersal from the basin of Yellow River in China^7^.

Curiously though, a previous mtDNA study of Zhangmu Sherpa reported considerable (8%–17%) South Asian genetic component among Sherpas^17^, but our results only show it in a minor frequency (0.34%–2.53%). Likely, this negligible gene flow from the Indian subcontinent towards the Sherpa is due to the natural barrier of the Himalayas, that have effectively firewalled the populations in the mountainous, low-oxygen environments that comprise the Sherpa homeland^5^,^18^,^19^.

Our observation of the large amount of East Asian genetic influence on the Nepalese population (Sherpas and non-Sherpas) suggests a recent unidirectional gene flow from East Asia to Nepal, not the opposite^20^,^21^. By the same token, our present data does not support the putative ancestry of the Sherpas and Han Chinese as progenitors of Tibetans^3^, and instead strongly supports that the Tibetans are ancestors of the Sherpas. While two Sherpa-specific mtDNA sub-lineages were found during our study, both are young in origin (<1500 years ago), and originated rather recently from the mother haplogroups of Tibetans, in line with recent historical records over the last 500 years ago that documents Sherpa migrations from eastern Tibet to the barren lands of Nepal’s Khumbu region by crossing the Nangpa La Pass (5,716m) in the Himalayan region (Fig. 1)^22^.

As for the Sherpa-specific sub-haplogroups, a previous mtDNA study^4^ proposed that the ND1 variants G3745A (C4a3b1) and T4216C (A15c1) may form the molecular basis of the Sherpas’ adaptation to hypoxic high-altitude. After conducting a selection test by calculating the non-synonymous vs. synonymous substitution (Ka/Ks) ratio of ND1, we did not observe a signal of selection (Ka/Ks = 1.0, Nei-Gojobori model). We also conducted Tajima’s D test using both the ND1 and HVS-I sequences, and no deviation from neutral expectation was detected (Tajima’s D = −1.271 for ND1, P > 0.1; Tajima’s D = −1.168 for HVS-I, P > 0.1), suggesting a neutral effect of these variants. Hence, the prevalence of the two Sherpa-specific mtDNA sub-lineages may better be explained by recent population expansion as reflected by the star-like phylogeny and their tip positions in the mtDNA phylogenetic tree (supplementary Figure S1). Accordingly, we propose that the ND1 variants may not have any direct role in hypoxic adaptation among Sherpas; indeed, our results suggest that the ancestors of Sherpas were already high altitude dwellers in the Tibetan plateau before they migrated from Tibet rather recently, which by extension predicts the same set of genes responsible for high-altitude previously identified in Tibetans (*e.g.* EPAS1 and EGLN1)^23^,^24^ should also be present among the Sherpas. In essence, the genetic adaption to high altitude in Sherpas were not likely to have developed locally in Nepal, but were instead inherent from their Tibetan ancestors who acquired the adaptive traits through their long stay (>18,000 years)^23^ in the harsh environment of the highland Tibetan plateau.

## Conclusion

Our mtDNA and Y-chromosome analyses of Sherpas indicate that Tibetans were the ancestral population of the Sherpas, and consequently that Sherpas likely acquired their high altitude adaptive features during their ancestors’ long-stay on the Tibetan plateau before their more recent migration towards Nepal. This recent origin of Sherpa-specific mtDNA sub-lineages may then not play any direct role in hypoxic adaptation. Ultimately, these genetic analyses both clarify long-standing debate on the origin of the Sherpas but also prompt the possibility that similar molecular mechanism for high altitude adaptation are shared between both Sherpas and Tibetans.

## Materials and Methods

### Sample collection

Blood samples of 2–5 ml were collected from 582 Sherpa individuals (350 from four villages of the Khumbu region of Nepal and 232 from Zhangmu Town of Tibet) (Fig. 1). We also sampled 90 non-Sherpa Nepalese from Solukhumbu district of Nepal (the lowland region with elevation <2000 m) who speak Tibeto-Burman languages. Volunteers were asked to reveal the ethnic affinity of their parents and grand-parents in an oral interview, and only volunteers whose parents and grandparents were reported to be Sherpas were included in this study. Sample collection was done randomly from individuals unrelated for at least three generations. All participants provided written informed consent prior to inclusion in the study. The protocols of this study were approved by the Internal Review Boards of the Kunming Institute of Zoology, Chinese Academy of Sciences and the Nepal Health Research Council, Kathmandu, Nepal. All methods were carried out in accordance with the approved guidelines.

### Y-chromosomal genotyping

The Y-chromosomal SNP markers were genotyped using methods similar to those described in our previous studies^10^,^25^,^26^, and paternal haplogroups were classified based on the up-to-date high-resolution Y-chromosomal phylogenetic tree^27^. The commonly used eight Y-chromosomal STR (short tandem repeat) markers (DYS19, DYS388, DYS389I, DYS389II, DYS390, DYS391, DYS392, and DYS393) were typed using fluorescence-labeled primers in an ABI 3130XL Genetic Analyzer (Applied Bio systems, USA). The nomenclature of Y-chromosomal STRs followed the process proposed by Butler *et al.* (2002)^28^.

### Mitochondrial DNA sequencing and genotyping

The mtDNA HVS-I (range:16024–16466), HVS-II (range:65–417) and a coding region (range:10,220–10,610) were sequenced following the protocol described previously^29^ and then we sequenced several other coding region sites for further confirmation used to define each individual within their respective mtDNA haplogroups. We then conducted amplicon sequencing of the entire mitochondrial genomes of 89 Sherpa samples using Illumina Miseq, including 79 individuals belonging to the two Sherpa-specific sub-haplogroups (32 A15c1 and 47 C4a3b1) and the other 10 individuals belonging to other haplogroups (5 M9a, 1 M70, 1 C4a1a1, 1 D4j1b and 1 A15c). The complete mtDNA was amplified via PCR of two overlapping fragments (9.3 kb and 9.2 kb)^30^. Amplicons were analyzed by agarose gel electrophoresis, individually purified via resin purification (Promega), quantified on a Bioanalyzer (Agilent Technologies 2100 Bioanlyzer) and equimolarly pooled. The Nextera DNA Sample Preparation guide (15027987) was followed for pooled amplicons to create indexed, paired-end libraries that were later sequenced on an Illumina Miseq (2 × 300 base reads) using the manufacturer’s protocol (Illumina, San Diego, CA). The resulting high quality sequence data from Miseq had an average coverage of 2000×. Sequence analysis was then done using mtDNA MSR Plug-In and the mtDNA Variant Analyzer provided by Illumina in Human mtDNA Genome Guide (15037958) for the Illumina Sequencing platform. (http://support.illumina.com/downloads/human_mtdna_genome_guide_15037958.html). The mtDNA variants were identified by comparing each sequence with the revised Cambridge Reference Sequence (rCRS)^31^, and haplogroups were assigned based on the mtDNA phylogenetic tree (Phylo Treemt Build 16,19 Feb 2014)^32^. Comparing a sample the variants produced by Miseq with those from Sanger sequencing confirmed that our MiSeq data were highly accurate and reliable.

### Data analysis

The Sherpa mtDNA phylogenetic tree was constructed based on the available whole genome mtDNA sequences of 165 Sherpa samples, including 76 Sherpa samples from previous studies^4^ and 89 samples obtained in this study. The Phylo Tree mt Build 16^32^ was followed for constructing the phylogenetic tree with exclusion of several mutations (309.1C (C), 315.1C, AC indels at 515–522, 16182C, 16183C, 16193.1C (C) and 16519). The median-joining network was constructed using NETWORK 4.6.1.0 (Fluxus Engineering)^33^ (Bandelt *et al.* 1999), and was constructed for the major haplogroups of both mtDNA and Y chromosome.

The coalescence times of the dominant mtDNA haplogroups were estimated using the ρ statistics^34^, and standard errors were calculated following Saillard *et al.*^35^. The complete mtDNA genome sequences were used for age estimations by excluding 309.1C (C), 315.1C, AC indels at 515–522, 16182C, 16183C, 16193.1C (C) and 16519). The mutation rate of 3,624 years per mutation was used for entire mtDNA genome to estimate the timeframe for the most recent common ancestor (TMRCA) of a haplogroup^36^.

To observe the genetic relationships of Sherpas with other populations, we constructed PCA maps based on both mtDNA and Y-Chromosome haplogroup frequencies as described previously using MVSP3.13 (Kovach Computing Services, Anglesey, UK)^37^. For the neutrality test, the ratio of non-synonymous vs. synonymous substitutions in the ND1 gene of Sherpas were analyzed using the Nei-Gojobori model^38^ in the MEGA5 package^39^. We also conducted Tajima’s D test using DnaSP Version 5^40^. We reconstructed the demographic history of Sherpas using BSP^41^ in BEAST 1.7.5^42^ using MCMC algorithms^43^. The HVS-I (16038–16462) sequences of available 661 Sherpa individuals were used for generating BSP. A clock rate of 1.784 × 10^−7^ substitutions per site per year^36^ was applied. MCMC sample was based on run of 5 × 10^8^ steps with sampling every 5,000 steps, and the initial 5 × 10^7^ were regarded as burn-in. The effective sample size values were in acceptable range (>100) for all the runs. Tracer 1.5 (http://tree.bio.ed.ac.uk/software/tracer) in the MCMC Trace analysis packages was used to visualize the BSP.

## Additional Information

Accession Numbers: The Genbank accession numbers for the 89 mtDNA whole-genome and 492 HVS-I sequences reported in this paper are KT213741 - KT213829 and KT213830 - KT214321 respectively. 

**How to cite this article**: Bhandari, S. *et al.* Genetic evidence of a recent Tibetan ancestry to Sherpas in the Himalayan region. *Sci. Rep.* **5**, 16249; doi: 10.1038/srep16249 (2015).

## Supplementary Material

Supplementary Information

## Figures and Tables

**Table 1:** Distribution of mtDNA haplogroups among Sherpas and Tibetans.

Haplogroup frequency (%)				
Populations	Sherpa (Nepal)	Sherpa (Tibet)	Sherpa (all)	Tibetan^5^
Sample Size	350	232	582	6,109
A	24.00	31.90	27.15	14.63
M9a	27.14	19.83	24.22	22.48
M8	28.00	17.24	23.71	7.71
M70	11.14	1.29	7.22	0.16
D	4.57	7.76	5.84	16.53
F	1.14	4.74	2.58	11.44
M13	0.57	3.45	1.72	4.22
U	0.29	3.02	1.37	1.65
M5	0.29	1.72	0.86	0.05
W	0.57	1.29	0.86	0.05
M3	0.29	1.72	0.86	0.00
G	0.57	0.86	0.69	8.22
M10	0.29	1.29	0.69	1.06
M62	0.00	1.29	0.52	2.35
M11a	0.29	0.86	0.52	0.79
H	0.00	1.29	0.52	0.26
M38	0.29	0.43	0.34	0.00
M61	0.29	0.00	0.17	0.75
M74	0.29	0.00	0.17	0.00
B	0.00	0.00	0.00	3.76
M*	0.00	0.00	0.00	0.79
TJ	0.00	0.00	0.00	0.72
R*	0.00	0.00	0.00	0.52
N*	0.00	0.00	0.00	0.47
M7	0.00	0.00	0.00	0.25
N10	0.00	0.00	0.00	0.25
M25	0.00	0.00	0.00	0.23
M20	0.00	0.00	0.00	0.20
N9a1	0.00	0.00	0.00	0.20
N11	0.00	0.00	0.00	0.18
M49	0.00	0.00	0.00	0.07
M12	0.00	0.00	0.00	0.02
Y	0.00	0.00	0.00	0.02
M12	0.00	0.00	0.00	0.02
