# Supplementary material for: Genetic evidence of a recent Tibetan ancestry to Sherpas in the Himalayan region
Source: Sci Rep. 2015 Nov 5;5:16249. doi: 10.1038/srep16249 (PMC4633682; doi:10.1038/srep16249)

Supplementary Information

Genetic evidence of a recent Tibetan ancestry to Sherpas in the Himalayan region

Sushil Bhandari[†,1,4], Xiaoming Zhang[†,1], Chaoying Cui[†2], Bianba[2], Shiyu Liao[1], Yi Peng[1], Hui Zhang[1], Kun Xiang[1], Hong Shi[1,5], Ouzhuluobu[2], Baimakongzhuo[2], Gonggalanzi[2], Shimin Liu[3], Gengdeng[3], Tianyi Wu[3], Xuebin Qi*[,1], Bing Su*[,1]

Supplementary Figure S1. The Y-STR network of four minor haplogroups in Sherpas.

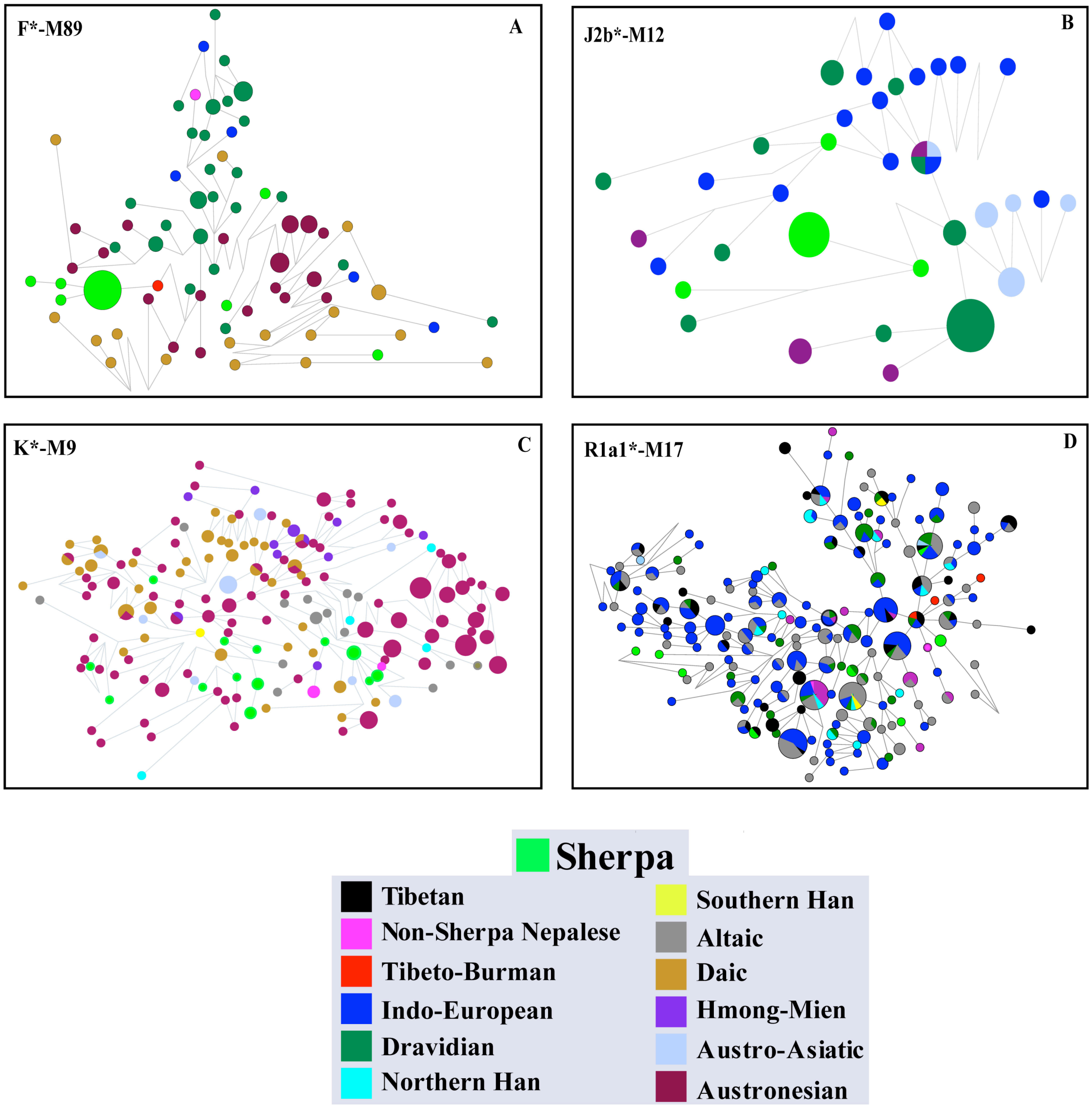

Supplementary Figure S2. The mtDNA phylogenetic tree based on 215 mitochondrial whole genome sequences, including 165 Sherpas (89 from present study and 76 from previous study) and 50 non-Sherpas (44 Tibetans, 4 Han Chinese and 2 Naxi from previous studies).

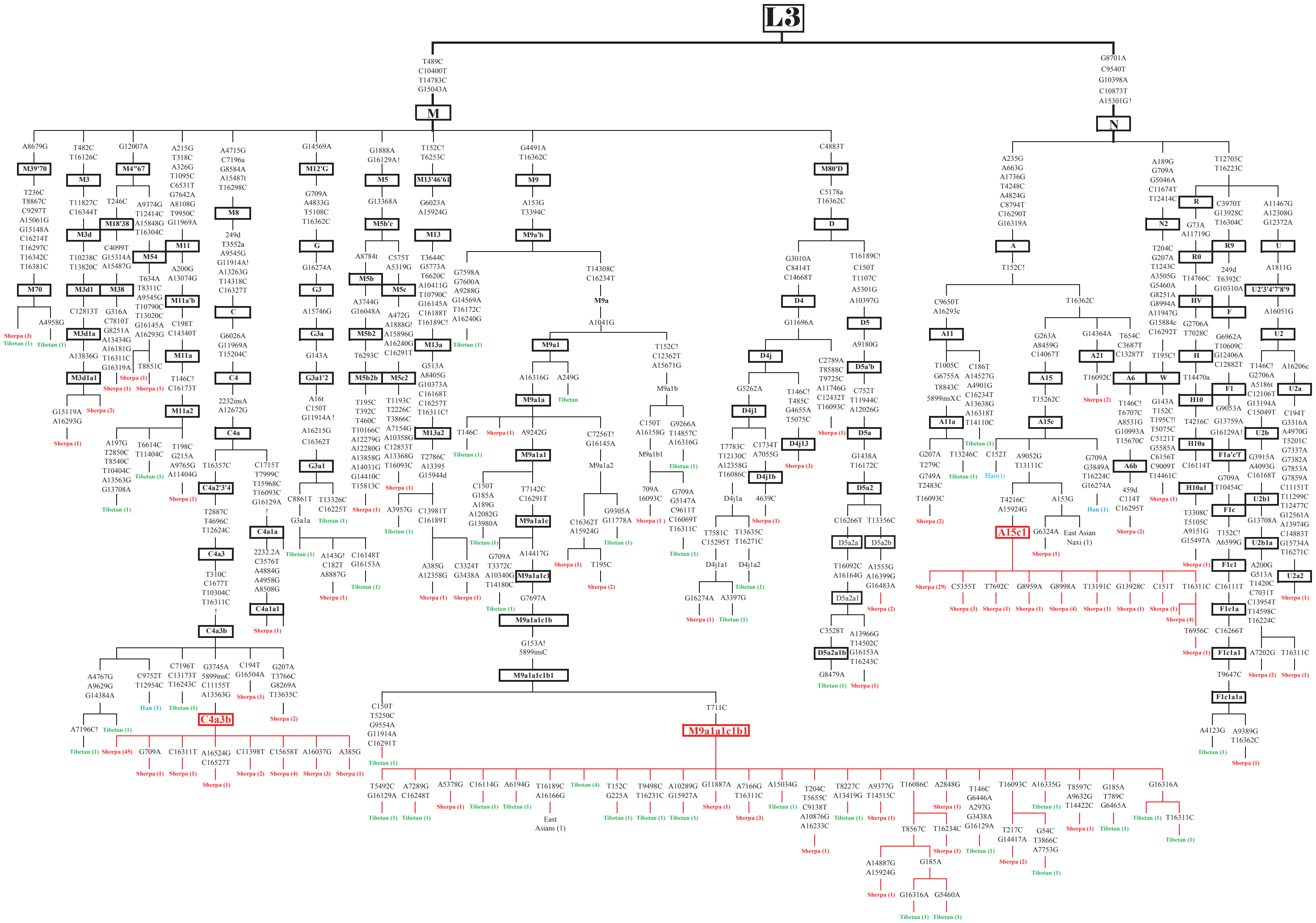

Supplementary Figure S3. The BSP plot showing population dynamics of Sherpas in history. A population bottleneck around 2,000 years ago was observed. The HVS-I sequences of Sherpas were used in the BSP analysis.

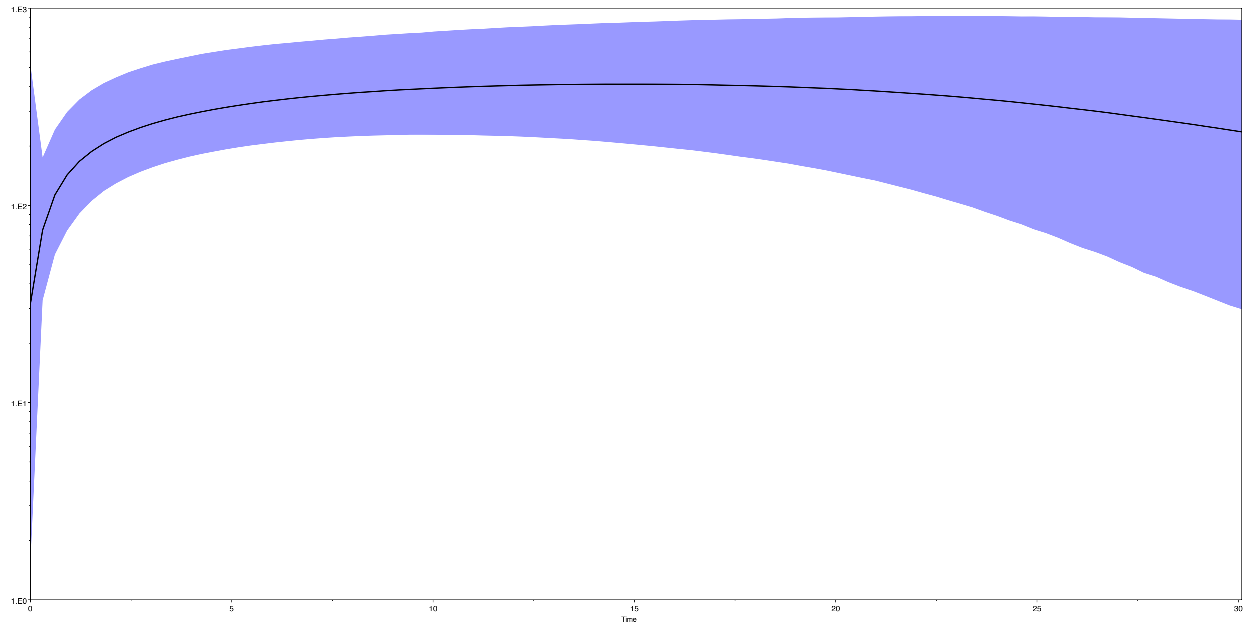

Supplement: Supplementary Information [file srep16249-s1.pdf]
